# Retrospective analysis on incidence and risk factors of early onset acute kidney injury after lung transplantation and its association with mortality

**DOI:** 10.1080/0886022X.2021.1883652

**Published:** 2021-03-18

**Authors:** Wen-Wen Du, Xiao-Xing Wang, Dan Zhang, Wen-Qian Chen, Xiang-Lin Zhang, Peng-Mei Li

**Affiliations:** Department of Pharmacy, China-Japan Friendship Hospital, Beijing, China

**Keywords:** Acute kidney injury, lung transplantation, tacrolimus, KDIGO, mortality

## Abstract

**Objectives:**

Acute kidney injury (AKI) is a common complication after lung transplantation (LTx) which is closely related to the poor prognosis of patients. We aimed to explore potential risk factors and outcomes associated with early post-operative AKI after LTx.

**Methods:**

A retrospective study was conducted in 136 patients who underwent LTx at our institution from 2017 to 2019. AKI was defined according to the Kidney Disease: Improving Global Outcomes (KDIGO) guideline. Univariate and multivariate analyses were conducted to identify risk factors related to AKI. The primary outcome was the incidence of AKI after LTx. Secondary outcomes were associations between AKI and short-term clinical outcomes and mortality.

**Results:**

Of the 136 patients analyzed, 110 developed AKI (80.9%). AKI was associated with higher baseline eGFR (odds ratio (OR) 1.01 (95% confidence interval (CI): 1.00–1.03)) and median tacrolimus (TAC) concentration (OR 1.15 (95% CI: 1.02–1.30)). Patients with AKI suffered longer mechanical ventilation days (*p* = .015) and ICU stay days (*p* = .011). AKI stage 2–3 patients had higher risk of 1-year mortality (HR 16.98 (95% CI: 2.25–128.45)) compared with no-AKI and stage 1 patients.

**Conclusions:**

Our results suggested early post-operative AKI may be associated with higher baseline eGFR and TAC concentrations. AKI stage 1 may have no influence on survival rate, whereas AKI stage 2–3 may be associated with increased mortality at 1-year.

## Introduction

Lung transplantation (LTx) remains the only choice for patients with end-stage lung disease who wish to improve life quality and prolong life span. However, despite progresses made in surgical techniques and immunosuppressive regimens, patients’ survival rates are still hampered by serious post-operative complications [1]. Acute kidney injury (AKI) is one of the most common complications with high incidence rates ranging from 39% to 69% [2]. It may be linked to other complications, including infection, bronchial dehiscence [3], sepsis [4], fluid overload [5], and possibly lead to increased short-term and long-term mortality [6].

Current knowledge on AKI is limited and sometimes contradictory, especially in the LTx population, regarding epidemiology, risk factors, and its relation with mortality [7]. Researchers have employed different definitions of AKI since 2005 [8], from risk, injury, failure, loss of kidney function and end-stage renal failure (RIFLE) criteria, acute kidney injury network (AKIN ) classification to KDIGO guideline, leading to a wide span of conclusions [9–11]. Potential risk factors ever reported included baseline glomerular filtration rate [12], transplant type [13], male sex [14], cyclosporine use [3], supratherapeutic tacrolimus (TAC) trough, ≥3 nephrotoxic drugs other than TAC and others [7,10]. Among these studies, study population concentrated in patients in the United States and Canada [3,15]. Only one study in Chinese population has been reported in 2014, and its population size was relatively small [16], leaving a major void in this area.

Therefore, our objectives in this retrospective study were: (1) analyzing the incidence and potential risk factors affecting the occurrence of AKI in a single Chinese center and (2) elucidating the relation between AKI and clinical outcomes.

## Materials and methods

### Study design and population

In this single center, observational, retrospective study, we included all patients who underwent LTx in China-Japan Friendship Hospital during the period between April 2017 and July 2019. The inclusion criteria were: (1) LTx for the first time and (2) age ≥18. The exclusion criteria were: (1) multiple organ transplantation; (2) survived less than seven days after transplantation; and (3) missing data.

The study was approved by the Ethics Committee of China-Japan Friendship Hospital (no. 2019-65-K45). All patients signed informed consent when undergoing LTx, in which they gave permission for use of anonymized medical data by scientists.

### Immunosuppressive regimens

Per protocols in our center, for induction therapy, patients received methylprednisolone or in combination with basiliximab. Methylprednisolone was given intraoperatively at a dose of 500–1000 mg. Basiliximab was given both before transplantation and on post-operative day (POD) 4 at a dose of 20 mg.

Maintenance immunosuppressive regimen consists of TAC, mycophenolate mofetil (MMF) and prednisone. TAC was given on POD1. Starting dose was 2 mg, twice daily, except those with exceptionally high or low body weight were given a personalized dose per attending physician’s judgment. Dose adjustments were based on whole-blood TAC trough concentration measurements. The target therapeutic range is 8–12 ng/mL in the first week. The initial dose of MMF was 1000 mg, twice daily and was adjusted according to area under the curve (AUC) measurements. Prednisone was given intravenously at 1 mg/(kg·d) during POD 1–3, and then switched to oral administration at 0.5 mg/(kg·d), and gradually tapered to 5–10 mg daily for long-term maintenance.

### AKI definition

AKI was determined and staged according to the ‘Kidney Disease: Improving Global Outcomes’ (KDIGO) Clinical Practice Guideline, based on serum creatinine (SCr) obtained from the transplantation date until seven days after transplantation [17]. According to the guideline, AKI was defined as any of the followings: (1) increase in SCr by ≥26.5 μmol/L within 48 h; (2) increase in SCr to ≥1.5 times baseline in the first seven PODs. AKI stages were determined as: (1) stage 1, SCr increase to 1.5–1.9 times baseline; (2) stage 2, SCr increase to 2.0–2.9 times baseline; (3) stage 3, SCr increase to ≥3.0 times baseline or to ≥353.6 μmol/L or initiation of renal replacement therapy (RRT). Baseline SCr was defined as SCr obtained prior to LTx. Recovery from an AKI episode was defined by return to no-AKI KDIGO class (<1.5 times baseline creatinine) at any time during the initial hospital stay after transplantation.

### Data collection

Patients’ electronic medical records were reviewed and data pertinent to AKI were collected. Baseline demographics (age, sex, height, and weight), pre-operative (pulmonary diagnosis, comorbidities, smoking history, baseline ALT, AST, RBC, hemoglobin, hematocrit, glucose, and SCr), intra-operative (transplant type, operation duration, ECMO support, and blood loss volume), and post-operative (ECMO support, TAC concentration, nephrotoxic drugs, mechanical ventilation duration, ICU stay, and hospital stay) data were obtained. eGFR were calculated using the modification of diet in renal disease (MDRD) equation [18].

Potential nephrotoxic drugs involved in patients’ regimen included: vancomycin, piperacillin tazobactam sodium, amikacin, trimethoprim, amphotericin B (inhalation), ganciclovir, acyclovir, rifampicin, and ethambutol.

### Outcome measures

The primary outcome was the incidence of AKI during POD1–POD7 after LTx. Secondary outcomes were short-term outcomes, including mechanical ventilation days, ICU stay days, and hospital stay days; and mortality, including 30-day and 1-year mortality. Hospital stay days were counted from transplantation day to first discharge from hospital.

### Statistical analysis

Distribution of data was assessed using the Shapiro–Wilk test. Descriptive statistics were expressed as mean ± standard deviation (SD) for normally distributed continuous variables or median (interquartile range, IQR) for non-normally distributed continuous variables. Categorical variables were presented as count (percentage). Differences between groups were analyzed by Student’s *t*-test or ANOVA for normally distributed continuous variables; Mann–Whitney’s *U* or the Kruskal–Wallis tests for non-normally distributed continuous variables, as appropriate. Pearson’s chi-square test or Fisher’s exact test was used to compare differences between groups in categorical variables. Potential risk factors with a *p*< .20 in univariate analysis were incorporated into multivariate logistic regression in a forward: LR stepwise fashion. Collinearity was assessed and model fit was assessed by the Hosmer–Lemeshow goodness-of-fit test. 30-day and 1-year survival rates stratified by AKI stages were analyzed by the Kaplan–Meier analysis and compared using the log-rank test. Associations of AKI stages and 30-day and 1-year mortality were assessed using the Cox proportional hazard models after adjusting for possible confounders, including baseline demographics, intra-operative and post-operative risk factors for AKI. Risk factors with *p*< .05 were considered statistically significant. Data were processed using Statistical Package for Social Science (SPSS) 19.0 (SPSS Inc., Chicago, IL).

## Results

### Incidence of AKI

During April 2017 and July 2019, 150 patients received LTx. From this study population, 11 were excluded due to missing data, two were excluded for death within 24 h, and one was excluded due to less than 18 years old, leaving 136 for final analysis. The median (IQR) age was 60 (54–64) years old, and 86.0% were male. The frequency of hypertension, diabetes mellitus, and hyperlipidemia before surgery was 20.6, 31.6, and 7.4%, respectively. The most common pulmonary diagnosis was interstitial lung disease (ILD; 71.3%), followed by chronic obstructive pulmonary disease (COPD; 16.9%), pulmonary hypertension (PH; 7.4%), others including bronchiolitis obliterans and bronchiectasis (3.7%) and cystic fibrosis (CF; 0.7%). The baseline eGFR was 137 ± 40 mL/min/1.73 m^2^.

**Figure 1.**
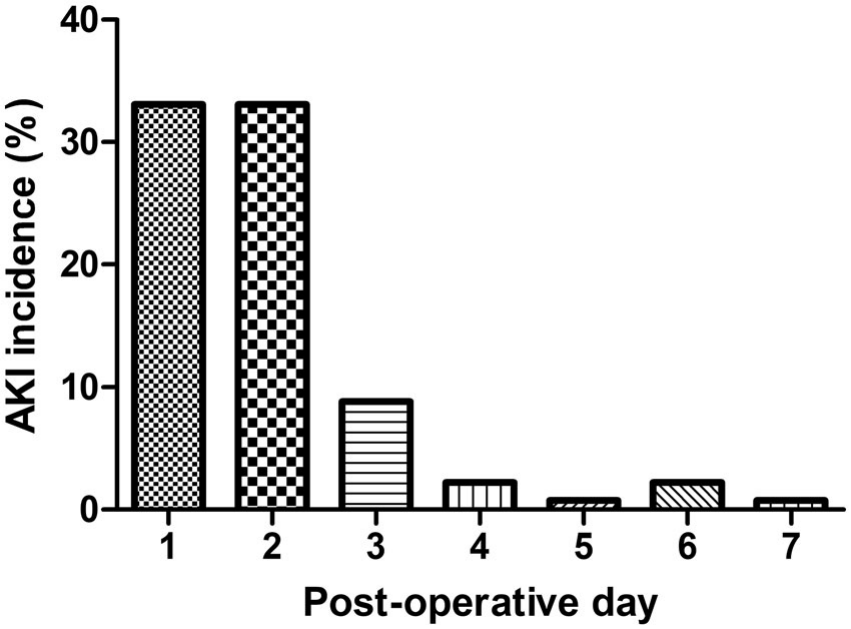
Incidence of AKI per day between day 1 and day 7 after lung transplantation.

Of the 136 patients analyzed, 110 experienced AKI (80.9%) during the first post-operative week, with the highest incidence on POD 1 and POD 2 (33.1%) (Figure 1). The incidence of AKI on POD 3–POD 7 was 8.8, 2.2, 0.7, 2.2, and 0.7%, respectively. AKI stage 1 occurred in 38 (27.9%), stage 2 in 32 (23.5%), and stage 3 in 40 (29.4%) of patients. Nineteen patients (14.0%) required RRT.

### Risk factors of AKI

Baseline demographic characteristics of the patients with AKI were similar to patients without AKI (Table 1). Pre-operative eGFR and SCr exhibited slight difference between no-AKI and AKI group; however, the difference was not statistically significant. In univariate analysis, only median TAC concentration was significantly associated with AKI occurrence (Table 2). In AKI group, median TAC concentration was 7.6 (4.7–11.0) ng/mL, while in no-AKI group it was 5.0 (2.4–7.8) ng/mL. Median TAC concentrations also showed ascending tendency across AKI stages.

**Table 1.** Baseline demographic characteristics of LTx patients by AKI status.

Parameters	No-AKI	AKI	*p* Value^a^	AKI stage			
				1	2	3	*p* Value^b^
*n* (%)	26 (19.1)	110 (80.9)		38 (27.9)	32 (23.5)	40 (29.4)	
Age	61 (54–66)	60 (54–64)	.496	61 (52–64)	62 (57–64)	59 (49–65)	.711
Male, *n* (%)	23 (88.5)	94 (85.5)	.934	31 (81.6)	26 (81.3)	37 (92.5)	.417
BMI, kg/m^2^	20.9 (3.9)	21.5(4.0)	.520	21.1(4.4)	22.1 (4.2)	21.4 (3.6)	.678
BSA, m^2^	1.8 (0.2)	1.8 (0.2)	.362	1.8 (0.2)	1.8 (0.2)	1.8 (0.2)	.556
Diagnosis, *n* (%)			.314				.392
COPD	7 (26.9)	16 (14.5)		3 (7.9)	4 (12.5)	9 (22.5)	
ILD	18 (69.2)	79 (71.8)		28 (73.7)	24 (75.0)	27 (67.5)	
PH	0 (0)	10 (9.1)		5 (13.2)	3 (9.4)	2 (5.0)	
CF	0 (0)	1 (0.9)		1 (2.6)	0 (0)	0 (0)	
Others	1 (3.8)	4 (3.6)		1 (2.6)	1 (3.1)	2 (5.0)	
Comorbidities, *n* (%)							
Hypertension	4 (15.4)	24 (21.8)	.466	6 (15.8)	5 (15.6)	13 (32.5)	.178
Diabetes mellitus	10 (38.5)	33 (30.0)	.404	13 (34.2)	12 (37.5)	8 (20.0)	.297
Hyperlipidemia	1 (3.8)	9 (8.2)	.731	1 (2.6)	4 (12.5)	4 (10.0)	.366
Smoking history, *n* (%)	22 (84.6)	80 (72.7)	.208	26 (68.4)	23 (71.9)	31 (77.5)	.483
SCr, μmol/L	60.2 (16.0)	54.5 (14.3)	.076	56.6 (15.8)	53.4 (11.9)	53.3 (14.6)	.227
eGFR, mL/min/1.73 m^2^	124 (33)	140 (41)	.056	134 (39)	137 (28)	149 (50)	.073
ALT, IU/L	20 (12–27)	18 (14–32)	.883	17 (11–24)	20 (15–34)	17 (14–35)	.329
AST, IU/L	20 (15–23)	20 (16–23)	.833	19 (15–23)	20 (18–40)	18 (16–22)	.098
RBC, ×10^12^ cells/L	7.0 (5.9–8.4)	6.7 (5.3–9.0)	.532	6.2 (5.0–8.9)	7.1 (6.0–9.1)	6.3 (5.2–8.8)	.317
Hemoglobin, g/L	131 (17)	137 (22)	.197	134 (21)	138 (20)	137 (25)	.524
Hematocrit, %	39.6 (5.1)	41.2 (6.6)	.267	40.9 (6.0)	41.1 (5.5)	41.5 (7.9)	.695
Glucose, mmol/L	4.2 (0.7)	4.5 (0.8)	.099	4.5 (0.7)	4.5 (0.7)	4.6 (0.9)	.358

LTx: lung transplantation; AKI: acute kidney injury; BMI: body mass index; BSA: body surface area; COPD: chronic obstructive disease; ILD: interstitial lung disease; PH: pulmonary hypertension; CF: cystic fibrosis; SCr: serum creatinine; eGFR: estimated glomerular filtration rate; ALT: alanine aminotransferase; AST: aspartate aminotransferase; RBC: red blood cells.

Categorical data are presented as number (%) (*n* (%)), continuous data presented as mean with standard deviation (mean (SD)) or median with inter-quartile range (med (IQR)), depending on variable distribution.

^a^ *p* value compared between no-AKI and AKI.

^b^ p value compared between different AKI stages and no-AKI.

**Table 2.** Intra-operative and post-operative parameters of LTx patients by AKI status.

				AKI stage			
Parameters	No-AKI	AKI	*p* Value^a^	1	2	3	*p* Value^b^
Transplant type, *n* (%)			.481				.208
Unilateral	18 (69.2)	68 (61.8)		25 (65.8)	23 (71.9)	20 (50.0)	
Bilateral	8 (30.8)	42 (38.2)		13 (34.2)	9 (28.1)	20 (50.0)	
Operation duration, min	225 (180–285)	255 (198–330)	.248	238 (210–338)	240 (180–330)	270 (180–334)	.670
Intra-operative ECMO support, *n* (%)	16 (61.5)	75 (68.2)	.517	29 (76.3)	21 (65.6)	25 (62.5)	.526
Loss of blood, mL	300 (100–500)	300 (200–500)	.368	300 (200–550)	300 (150–400)	300 (200–575)	.546
Basiliximab use, *n* (%)	10 (38.5)	45 (40.9)	.819	15 (39.5)	11 (34.4)	19 (47.5)	.712
Post-operative ECMO support, *n* (%)	15 (57.7)	76 (69.1)	.267	29 (76.3)	21 (65.6)	26 (65.0)	.456
Median TAC concentration, ng/mL	5.0 (2.4–7.8)	7.6 (4.7–11.0)	.011	5.9 (3.7–8.8)	7.9 (4.9–10.9)	9.0 (6.5–13.9)	.001
Nephrotoxic drugs ≥ 6, *n* (%)	6 (23.0)	38 (34.5)	.261	9 (23.7)	10 (31.3)	19 (47.5)	.088

LTx: lung transplantation; AKI: acute kidney injury; ECMO: extracorporeal membrane oxygenation; TAC: tacrolimus.

Categorical data are presented as number (%) (*n* (%)), continuous data presented as mean with standard deviation (mean (SD)) or median with inter-quartile range (med (IQR)), depending on variable distribution.

^a^ *p* value compared between no-AKI and AKI.

^b^ *p* value compared between different AKI stages and no-AKI.

Five variables, including baseline SCr, eGFR, hemoglobin, glucose, and median Tac concentration were further assessed in multivariate model (Table 3). Higher baseline eGFR (odds ratio (OR) 1.01 (95% confidence interval (CI): 1.00–1.03)) and median TAC concentrations (OR 1.15 (95% CI: 1.02–1.30)) post-transplantation were associated with associated with increased risk of AKI.

**Table 3.** Multivariate analysis for risk factors of AKI.

Covariate	Univariate		Multivariate	
	OR (95% CI)	*p* Value	OR (95% CI)	*p* Value
SCr, μmol/L	0.98 (0.95–1.00)	.080		
eGFR, mL/min/1.73 m^2^	1.01 (1.00–1.03)	.056	1.01 (1.00–1.03)	.049
Hemoglobin, g/L	1.01 (0.99–1.04)	.196		
Glucose, mmol/L	1.67 (0.91–3.07)	.099		
Median TAC concentration, ng/mL	1.14 (1.02–1.29)	.024	1.15 (1.02–1.30)	.022

CI: confidence interval; AKI: acute kidney injury; SCr: serum creatinine; eGFR: estimated glomerular filtration rate; TAC: tacrolimus.

*p* Value for the Hosmer–Lemeshow goodness-of-fit test was .601.

### Short-term outcomes

Of our study population, the median (IQR) mechanical ventilation days was 2 (1–3), median (IQR) ICU stay days was 4 (3–5) and median (IQR) hospital stay days was 44 (30–60). Table 4 shows the post-operative short-term outcomes in no-AKI patients and AKI patients. AKI patients had longer mechanical ventilation days and ICU stay days compared with no-AKI group. No statistically significant difference was observed in hospital stay days (Table 4).

**Table 4.** Post-operative outcomes by AKI status.

				AKI stage			
Parameters	No-AKI	AKI	*p* Value^a^	1	2	3	*p* Value^b^
IMV days, d	1 (1–2)	2 (1–3)	.015	2 (1–2)	2 (1–2)	2 (2–8)	.002
ICU stay, d	3 (2–4)	4 (3–6)	.011	4 (3–5)	4 (3–5)	6 (3–10)	.009
Hospital stay, d	42 (34–56)	45 (30–63)	.605	42 (32–56)	46 (29–92)	46 (24–72)	.812

AKI: acute kidney injury; IMV: invasive mechanical ventilation; ICU: intensive care unit.

Data presented as median with inter-quartile range (med (IQR)), depending on variable distribution.

^a^ *p* value compared between no-AKI and AKI.

^b^ *p* value compared between different AKI stages and no-AKI.

When comparing mechanical ventilation days between different stages, we found AKI stage 3 patients suffered longer duration than no-AKI patients and AKI stage 1 (*p* = .002 and *p* = .034, respectively; Bonferroni’s correction). Also, stage 3 patients spent more days in ICU than no-AKI patients (*p* = .006; Bonferroni’s correction).

### Survival outcomes

**Figure 2.**
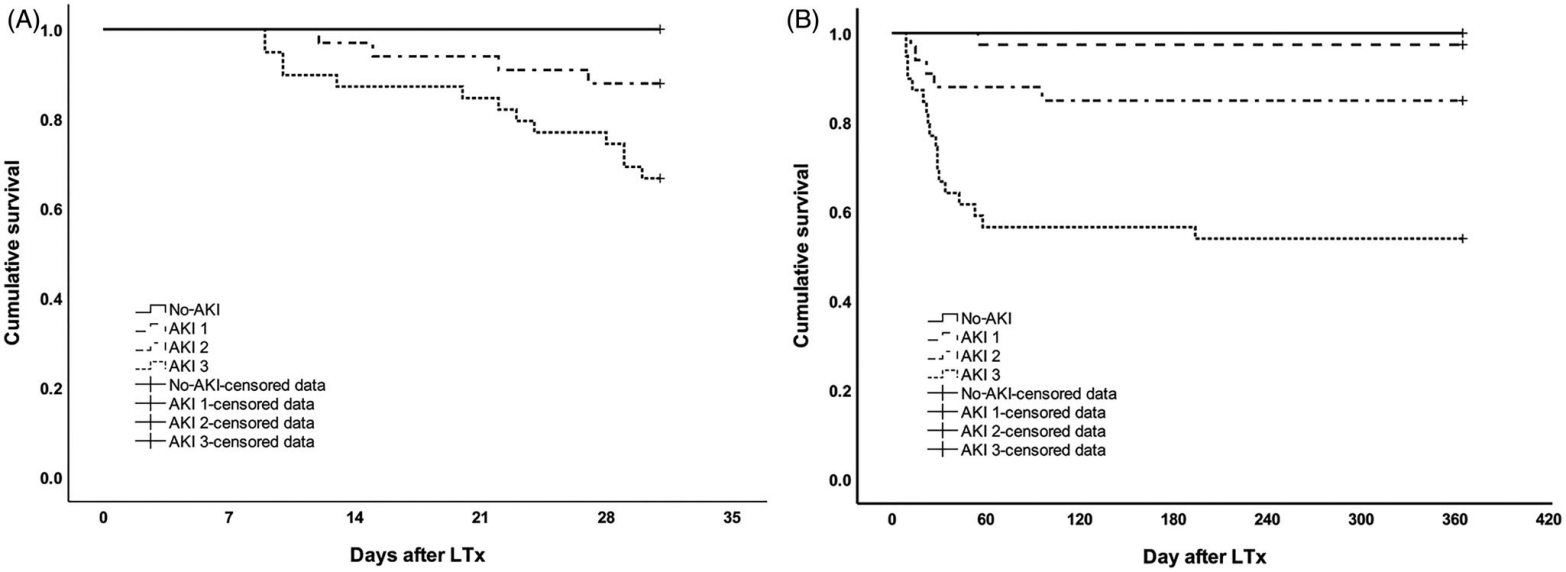
Kaplan–Meier’s survival curves stratified by AKI stages and no-AKI at (A) 30-day and (B) 1-year.

Thirty-day and 1-year survival rates of the study population after LTx were 87.5 and 82.4%, respectively. The Kaplan–Meier analysis showed 30-day survival rate was 100.0, 100.0, 87.5, and 67.5% in no-AKI, AKI stages 1, 2, and 3, respectively (no-AKI versus AKI stage 2, *p* = .065; no-AKI versus AKI stage 3, *p* = .002); and 1-year survival rate was 100.0, 97.4, 84.4, and 55.0%, respectively (no-AKI versus AKI stage 1, *p* = .408; no-AKI versus AKI stage 2, *p* = .037; no-AKI versus AKI stage 3, *p* = 9.7 × 10^−5^) (Figure 2).

According to Kaplan–Meier’s analysis, no statistically significance in survival rates between no-AKI and AKI stage 1 was observed. Hence, we combined no-AKI and AKI stage 1 into one group for further analysis. From the Cox proportional hazard model (adjusting for potential confounders, including baseline demographics, intra-operative and post-operative risk factors for AKI), severe AKI (stages 2 and 3) in the first post-operative week was found to be associated to increased risk of 1-year mortality (HR 16.98 (95% CI: 2.25–128.45)), but not 30-day mortality (Tables S1 and S2).

## Discussion

We conducted a retrospective study in Chinese LTx patients to elucidate the incidence, risk factors of AKI and its association with clinical outcomes. We drew three major conclusions from this study: (1) AKI has a high occurrence in the first post-operative week, (2) it relates to baseline eGFR and TAC concentration, and (3) it is associated with increased 30-day and 1-year mortality.

The overall incidence of AKI in our study population was 80.9%, which was high in comparison with previous studies [10,14,19]. It may be due to the following reasons: (1) different AKI definitions and (2) population demographics. A meta-analysis in LTx patients summarized data published between 2005 and 2019: the incidence rate of AKI was estimated to be 49% (95% CI: 38.3–59.8%), 55.5% (95% CI: 45.2–65.4%), and 53% (95% CI: 38.2–67.3%) by RIFLE, AKIN, and KDIGO criteria, respectively. KDIGO criteria combines the concept of relative baseline SCr change within seven days in RIFLE criteria and a small increase in SCr within 48 h in AKIN criteria, therefore, it has higher sensitivity and could theoretically recognize more AKI patients [17,20]. The highest incidence rate classified by KDIGO criteria was 68.8%, reported by Fidalgo et al. [3], which was still lower than ours. We analyzed our population demographics, and found the majority of LTx patients were elderly male patients (Figure S1). Bennett et al. reported male gender was related to higher risk of AKI [14], and attributed it partly to the data reported by the International Society for Heart and Lung Transplantation (ISHLT) registry claiming that male gender was associated with worse lung transplant outcomes [21]. Also, he mentioned AKI patients were slightly older than no-AKI patients, which was in accordance with conclusion drew by George et al. [13] and hence the high incidence of AKI may be partly attributed to the characteristic of our population demographics.

One major finding in our study was higher baseline eGFR contributed to AKI after LTx, which was counterintuitive, and contradicted by many previous studies [8,12,22]. Similar results reported were explained by a higher proportion of cystic fibrosis patients [8,23]; however, since only one case of cystic fibrosis was found in our study, this explanation does not seem to work for us. Study by Navis et al. also showed an inverse relationship between baseline GFR and post-operative renal function at 1 month [24]. He concluded patients with renal impairment before LTx would have less pronounced decline in renal function after surgery. However, most patients in our study (91.2%) had eGFR values over 90 mL/min/1.73 m^2^, which indicates a normal renal function and hence is not consistent with the reported population setting in Navis’ study.

One possible mechanism behind the link between higher baseline eGFR and AKI is glomerular hyperfiltration (GH). Glomerular hyperfiltration, described as an absolute increase in GFR, occurs in various clinical conditions including diabetes, obesity, pregnancy, and critically ill patients [25,26]. Hyperfiltration state may indicate kidneys have little reserve capacity, thus intolerable to nephrotoxic insults and prone to incidence of AKI. Sandery et al. also demonstrated in their study that the elevated baseline eGFR was a significant risk factor of AKI in children receiving nephrotoxic drugs, and attributed the pathogenesis to GH [27].

TAC concentration was associated with post-operative AKI in our study, and a gradient increase with worsening AKI severity was observed (Table 2). The acute nephrotoxicity of TAC may be due to vasoconstriction of glomerular afferent arterioles when concentration is high [28,29]. Till now, only two studies elucidated the relationship between TAC concentration and post-operative AKI in LTx patients [10,30]. Similar to Sikma et al.’s study, our study also found a small proportion of AKI patients were supratherapeutic (7.9, 18.7, and 30.0% for AKI stages 1, 2, and 3, respectively). This conclusion suggests even routine whole-blood concentration measurements of TAC were not supratherapeutic, patients may still face the risk of developing AKI. Sikma et al. proposed using unbound plasma concentration in lieu of whole-blood concentration, which may be a more sensitive marker of nephrotoxicity. However, currently, most centers routinely test whole-blood TAC concentration, either by immunoassays or by LC–MS/MS, to guide dosing [31]. New therapeutic drug monitoring (TDM) approaches focusing on intracellular or tissue TAC concentration maybe a valuable option in predicting patients’ outcomes in the future [31,32].

AKI patients suffered longer postoperative mechanical ventilation duration and longer ICU stay but did not alter hospital stay days in our study. Correlation between mechanical ventilation and AKI has been reported in previous studies [3,16,33]. Since this is a retrospective study, we could not determine the causal relationship between AKI and mechanical ventilation. Koyner and Murray described a ‘deleterious bidirectional relationship’ between AKI and acute lung injury [34]. This pathological lung–kidney crosstalk, including perioperative lung ischemia/reperfusion and ventilator-induced lung injury, could possibly precipitate the onset of AKI after LTx [35]. Also, AKI may aggravate patients’ conditions and lead to weaning failure [36].

Regardless of staging severity, the association between AKI and mortality has been addressed in many studies [8,14,33]. We investigated 30-day and 1-year mortality and found the survival rates were significantly different between AKI and no-AKI patients (Figure S2). But when further stratified by stages, no significant difference was found between AKI stage 1 and no-AKI patients at both 30-day and 1-year (Figure 2). The 2017 consensus report on acute kidney disease and renal recovery of the Acute Disease Quality Initiative (ADQI) 16 Workgroup summarized that recovery from AKI within 48–72 h after onset is associated with better outcomes than longer durations of AKI [6]. In our study, 52.6% of stage 1 patients (*n* = 20) recovered from AKI within 72 h (Table S3), whereas 6.2% of stage 2 (*n* = 2) and no patients from stage 3 were recovered. Hence, this result could partly explain the finding in Figure 2. Similar to our study, Xue et al. also reported only severe AKI group (AKIN stages 2 and 3) was associated with higher long-term mortality rates [16]. Moreover, Calabrese et al. directly used KDIGO stages 2 and 3 as the definition of AKI in their study [37]. These findings were consistent with our conclusion that AKI stages 2 and 3 were related increased 1-year mortality.

The characteristic of our population demographics made this study unique: (1) we had less cystic fibrosis patients compared with patients from previous studies; (2) our patients were mainly elderly male patients. Meanwhile, several notable limitations exist in our study. First, this is a retrospective study and not all potential risk factors for AKI were collected due to missing data on electronic medical records, including ischemic time, blood transfusions, vasopressors requirement, genotypes, and other factors ever reported. Second, we did not include urine output data in AKI definition or fluid volume data in statistical analysis because we were not able to acquire the data. Fluid status is a significant factor to consider when interpreting AKI incidence and clinical outcomes. To protect allograft function, patients are maintained on negative fluid balance early after LTx, possibly leading to decreased renal perfusion [38]. Meanwhile, fluid overload and oliguria have been reported to be associated with worse clinical outcomes, including longer mechanical ventilation days, ICU stay days, and increased mortality in both no-AKI and AKI patients [5,39]. Hence, the influence of fluid balance following LTx on clinical outcomes needs to be clarified in further investigation. Third, as we mentioned above, our study population were concentrated in male, elderly patients, which may not reflect the reality in female and younger patients. Future studies, especially multi-center, prospective study on large-population are needed to elucidate risk factors pertain to AKI after LTx.

In conclusion, our single-center experience shows that LTx patients have a high incidence of AKI after surgery. Factors including baseline eGFR and TAC concentrations are associated with AKI onset. AKI patients suffered longer duration of mechanical ventilation and ICU stay. Approximately, half patients of AKI stage 1 could recover from AKI and AKI stage 1 did not significantly affect survival rate, whereas AKI stages 2 and 3 are associated with increased mortality. This finding may imply that severe AKI (stages 2 and 3) at early post-operative period identifies higher mortality risk and patients may deserve closer attention.

## Supplementary Material

Supplemental Material

## Abbreviations

ADQI: acute disease quality initiative
AKI: acute kidney injury
AKIN: acute kidney injury network
ALT: anine aminotransferase
AST: aspartate aminotransferase
AUC: area under curve
BMI: body mass index
BSA: body surface area
CF: cystic fibrosis
CI: confidence interval
COPD: chronic obstructive disease
ECMO: extracorporeal membrane oxygenation
eGFR: estimated glomerular filtration rate
GH: glomerular hyperfiltration
ICU: intensive care unit
ILD: interstitial lung disease
IMV: invasive mechanical ventilation
IQR: interquartile range
ISHLT: international society for heart and lung transplantation
KDIGO: kidney disease = improving global outcomes
LTx: lung transplantation
MDRD: modification of diet in renal disease
MMF: mycophenolate mofetil
OR: odds ratio
PH: pulmonary hypertension
POD: post-operative day
RBC: red blood cells
RIFLE: risk, injury, failure, loss of kidney function and end-stage renal failure
RRT: renal replacement therapy
SCr: serum creatinine
SD: standard deviation
TAC: tacrolimus
TDM: therapeutic drug monitoring
