# Supplementary material for: Retrospective analysis on incidence and risk factors of early onset acute kidney injury after lung transplantation and its association with mortality
Source: Ren Fail. 2021 Mar 18;43(1):535–42. doi: 10.1080/0886022X.2021.1883652 (PMC7993381; doi:10.1080/0886022X.2021.1883652)

Table S1. Univariate and multivariate COX analysis for 30-day mortality

	Univariate		Multivariate	
**Covariate**	**HR (95% CI)**	***p* value**	**HR (95% CI)**	***p* value**
Age	1.02 (0.97–1.07)	0.490		
Sex	0.82 (0.19–3.57)	0.788		
BMI, kg/m^2	1.06 (0.94–1.19)	0.371		
BSA, m^2	1.77 (0.13–24.62)	0.672		
Diagnosis				
COPD	Reference	0.999		
ILD	1.04 (0.30–3.63)	0.957		
PH	0.76 (0.08–7.26)	0.808		
CF	0.00 (0.00–.)	0.991		
Others	0.00 (0.00–.)	0.981		
Comorbidities				
Hypertension	1.62 (0.57–4.60)	0.364		
Diabetes mellitus	1.17 (0.28–1.67)	0.759		
Hyperlipidemia	2.95 (0.85–10.28)	0.089	1.16 (0.26–5.14) [a]	0.841
SCr, µmol/L	0.99 (0.95–1.02)	0.414		
eGFR, ml/min/1.73 m^2	1.00 (0.99–1.02)	0.364		
ALT, IU/L	0.98 (0.95–1.02)	0.353		
AST, IU/L	0.98 (0.94–1.04)	0.549		
RBC, $\times 10^{12}$ cells/L	1.18 (1.03–1.35)	0.015	1.10 (0.96–1.27)	0.162
Hemoglobin, g/L	1.01 (0.98–1.03)	0.594		
Hematocrit, %	1.01 (0.94–1.09)	0.713		
Glucose, mmol/L	1.16 (0.64–2.09)	0.625		
Transplant type				
Unilateral	Reference			
Bilateral	1.23 (0.47–3.24)	0.670		
Operation duration, min	1.00 (1.00–1.01)	0.536		
Intra-operative ECMO support	1.17 (0.41–3.32)	0.767		
Loss of blood, ml	1.00 (1.00–1.00)	0.885		
Basiliximab use	1.30 (0.50–3.37)	0.588		
Post-operative ECMO support	1.64 (0.53–5.02)	0.389		
Median TAC concentration, ng/ml	1.14 (1.08–1.20)	<0.001	1.08 (1.01–1.16)	0.027
Nephrotoxic drugs≥6, n (%)	1.52 (0.58–4.01)	0.392		
AKI stage 2-3	65.50 (1.32–3241.24)	0.036	2.33×10^{-5}(0.00–.)	0.927

Abbreviations: CI, Confidence Interval; HR, hazard ratio; AKI, Acute Kidney Injury; BMI, Body Mass Index; BSA, Body Surface Area; COPD, Chronic Obstructive Disease; ILD, Interstitial Lung Disease; PH, Pulmonary Hypertension; CF, Cystic Fibrosis; SCr, Serum Creatinine; eGFR, estimated Glomerular Filtration Rate; ALT, Anine aminotransferase; AST, Aspartate aminotransferase; RBC, Red Blood Cells; ECMO, Extracorporeal Membrane Oxygenation; TAC, tacrolimus.

Table S2. Univariate and multivariate COX analysis for 1-year mortality

	Univariate		Multivariate	
**Covariate**	**HR (95% CI)**	***p* value**	**HR (95% CI)**	***p* value**
Age	1.01 (0.97–1.05)	0.702		
Sex	0.55 (0.13–2.33)	0.416		
BMI, kg/m^2	1.02 (0.92–1.12)	0.723		
BSA, m^2	0.96 (0.10–8.88)	0.974		
Diagnosis				
COPD	Reference	0.762		
ILD	0.59 (0.23–1.52)	0.273		
PH	1.13 (0.28–4.51)	0.864		
CF	0.00 (0.00–.)	0.990		
Others	0.00 (0.00–.)	0.977		
Comorbidities				
Hypertension	1.32 (0.52–3.32)	0.559		
Diabetes mellitus	0.89 (0.37–2.14)	0.792		
Hyperlipidemia	2.04 (0.61–6.83)	0.249		
SCr, µmol/L	0.99 (0.96–1.02)	0.529		
eGFR, ml/min/1.73 m^2	1.00 (0.99–1.01)	0.465		
ALT, IU/L	1.00 (0.98–1.02)	0.907		
AST, IU/L	1.00 (0.97–1.04)	0.825		
RBC, ×10^{12} cells/L	1.12 (0.99–1.26)	0.077		
Hemoglobin, g/L	1.01 (1.00–1.03)	0.179		
Hematocrit, %	1.03 (0.98–1.09)	0.272		
Glucose, mmol/L	1.29 (0.80–2.07)	0.291		
Transplant type				
Unilateral	Reference			
Bilateral	1.26 (0.56–2.84)	0.575		
Operation duration, min	1.00 (1.00–1.01)	0.280		
Intra-operative ECMO support	0.68 (0.30–1.54)	0.361		
Loss of blood, ml	1.00 (1.00–1.00)	0.845		
Basiliximab use	1.50 (0.67–3.34)	0.322		
Post-operative ECMO support	0.84 (0.37–1.93)	0.686		
Median TAC concentration, ng/ml	1.13 (1.07–1.19)	<0.001	1.10 (1.03–1.17)	0.003
Nephrotoxic drugs≥6, n (%)	2.20 (0.99–4.90)	0.053	1.70 (0.75–3.84)	0.203
AKI stage 2-3	24.46 (3.30–181.19)	0.002	16.98 (2.25–128.45)	0.004

Abbreviations: CI, Confidence Interval; HR, hazard ratio; AKI, Acute Kidney Injury; BMI, Body Mass Index; BSA, Body Surface Area; COPD, Chronic Obstructive Disease; ILD, Interstitial Lung Disease; PH, Pulmonary Hypertension; CF, Cystic Fibrosis; SCr, Serum Creatinine; eGFR, estimated Glomerular Filtration Rate; ALT, Anine aminotransferase; AST, Aspartate aminotransferase; RBC, Red Blood Cells; ECMO, Extracorporeal Membrane Oxygenation; TAC, tacrolimus.

Table S3. AKI recovery status after LTx

		Recovery	
**AKI stages**	**Total (n)**	**48 h (n, %)**	**72 h (n, %)**
Stage 1	38	13 (34.2)	20 (52.6)
Stage 2	32	0	2 (6.2%)
Stage 3	40	0	0

Figure S1. Age distribution among study population

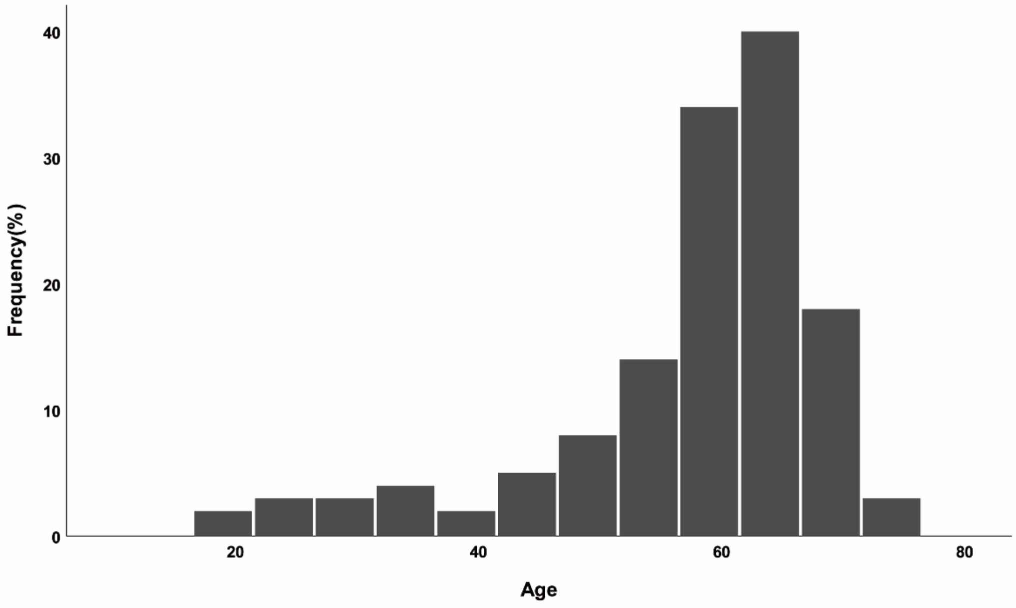

Figure S2. (A) 30-day and (B) 1-year survival stratified by AKI status

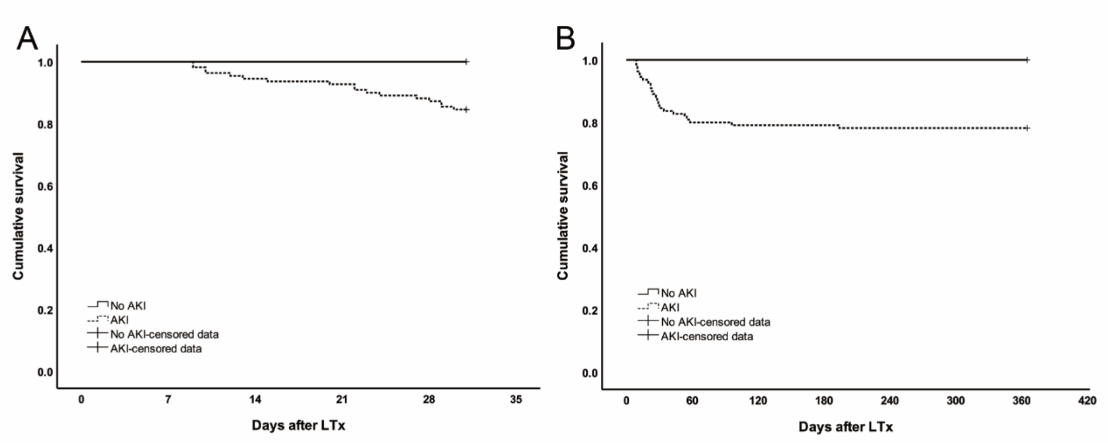

Supplement: Supplemental Material [file IRNF_A_1883652_SM3205.pdf]
